# Supplementary material for: Delayed detection of leprosy cases: A systematic review of healthcare-related factors
Source: PLoS Negl Trop Dis. 2022 Sep 6;16(9):e0010756. doi: 10.1371/journal.pntd.0010756 (PMC9481154; doi:10.1371/journal.pntd.0010756)
Supplement: Attachment — Submitted filename: The reply letter.docx [file pntd.0010756.s003.docx]

Dear Dr Alberto Novaes Ramos Jr,

Please find below our responses to the feedback of the reviewers as well as the changes we made to our manuscript.
With best wishes,
on behalf of all authors,
Yudhy Dharmawan

**Methods**
Reviewer #1: A well written article

Reviewer #2: -Are the objectives of the study clearly articulated with a clear testable hypothesis stated?
Yes. The aim of this systematic review was to identify healthcare factors related to delays in case detection in leprosy.
-Is the study design appropriate to address the stated objectives?
No. The framework of healthcare factors related to case detection delay(Figure 2.) is confusing. The authors classified healthcare factors related to case detection delay as macrolevel factors, microlevel factors and intermediate factors. It is innovative, however, it is difficult to classify them. For example, in macrolevel factors: iii) health service organisation and management (classified as factors underlying the microlevel factors). It is contradictory. The authors believed that poor geographical access is macrolevel factor, but lack of specialists caused by geographical barriers is microlevel factor. The authors believed that decentralisation and integration is macrolevel factor but the limited number of referral centers is microlevel factor. What is intermediate factor? it is also confusing. The problem analysis tree is not clear. In my opinion, the quality of healthcare is ususal evaluated by availability and accessibility, and then find their related factors.
***Reply :***

*We have revised Figure 2 to avoid confusion for the readers. Now, we use the term Structural factors instead of Macrolevel factors and Health service factors instead of Microlevel factors ( Please see Figure 2).
In drafting Figure 2, we collected all health care factors as mentioned in the studies and described all relations of these factors to case detection delay. When possible we report relations and associations as identified in the studies (indicated by references numbers) and otherwise we indicate inferred relations. According to our review of the included studies and considering the pathway revealed in each study, we aimed to categorize the factors that are related to case detection delay in three groups to make the pathway clear to all readers. Structural factors are related to health service factors, which, with or without intermediate factors, contribute to leprosy case detection delay. Intermediate factors occur in a causal pathway between health service factors and case detection delay. These factors give variation in the case detection delay.*

*To make it clear, we have revised the manuscript as below:*

*-Rows 31-38 :*

*We identified three categories of healthcare factors related to delayed case. 1)* *Structural factors, such as i) financial and logistic issues, and geographical circumstances (which we classified as barriers); ii) Health service organisation and management including the level of decentralisation (classified as facilitators). 2) Health service factors, such as problems or shortages involving referral centers, healthcare personnel, and case-detection methods. 3) Intermediate factors, such as misdiagnosis, higher numbers of consultations before diagnosis, and inappropriate healthcare services visited by people with leprosy.*

*-Rows 54-56:*

*Detection delay was further influenced by geographical circumstances, financial and logistic issues, health-service organisation and management including the level of the decentralisation of healthcare programs.*

*-Rows 180-184 :*

1. ***Structural factors***

*Structural* *factors are basic health-service-related factors that contribute to delays in case detection. They include i) financial and logistic issues, and geographical circumstances (which we classified as barriers); ii) health service organisation and management including of the level of decentralisation of healthcare services (classified as facilitators).*

*-Rows 211-213 :*

2. ***Health Services factors***

*Our review found many Health service factors related to healthcare and human resources that contributed to delayed case detection (Fig 2).*

*-Rows 261-262:*

*We identified three intermediate factors that contributed to delayed case detection (Fig 2). These factors occur in a causal pathway from health service factors to case detection delay.*

*-Rows 309-315:*

*This systematic review distinguished three categories of healthcare factor related to delayed case detection of leprosy: 1) Structural factors, which included geographical circumstances, financial and logistic issues, and health-service organisation and management including the level of decentralisation; 2) Health service factors, which included healthcare personnel, case-detection methods, and lack of referral centers or issues with them; and 3) Intermediate factors such as misdiagnosis, a high number of consultations, and inappropriate healthcare services visited by people with leprosy.*

-Is the population clearly described and appropriate for the hypothesis being tested?
Yes.
-Is the sample size sufficient to ensure adequate power to address the hypothesis being tested?
Yes.
-Were correct statistical analysis used to support conclusions?
Yes.
-Are there concerns about ethical or regulatory requirements being met?
Yes.
Reviewer #3:
In row 98 – did the authors searched the grey literature? If not, please provide the reason why.
***Reply*** *:*
*We did not search the grey literature. We focused on scientific evidence and therefore only included empirical studies that have been published in peer-reviewed journals.*

In rows 98-106: It is important to refer with details to the PICO\PECO criteria (population, exposure, comparison group and outcome) when describing the selection criteria in the text. For example, if delays are measured in days, the authors used any cut-off point to define delay or just used delay as a continuous variables? Would both types be included in the SR? In addition, there if no information on the comparison group or the exposure.

***Reply*** *:*

*We thank the reviewer for this feedback and have revised our text. We added the sentences in rows 102-108 :*

*To identify factors determining delayed detection, we used PICO ( Population, Intervention/Exposure, Comparison and Outcome) to make structured inclusion and exclusion criteria. Population: leprosy patients in leprosy high and low endemic countries were included. Exposure: Health care services factors.* *Comparison: we included all studies which presented case detection delay as expressed in time periods of months and as cases categorised as G2D without comparing the health care services factors that contributed to them. Outcome: the disability in leprosy*

Rows 105-106- Other narrative or systematic reviews were included in the SR?

***Reply*** *:*

*No, we only included the original empirical studies*

*Please, see rows 110-111 :*

*We included original empirical leprosy-related studies published in English between January 1, 2000, and January 31, 2021.*

In rows 116-124– The authors do not report metanalysis. This was consider in the study and it was not possible or it was not considered in the first place? In addition, please state that a narrative review of the literature was performed.

***Reply*** *:*

*We only did a narrative analysis. A meta-analysis was not feasible due to the variation in the methods as applied in the included studies.*

*We now have added the sentence in rows 131-132 :*

*We conducted a narrative analysis. A meta-analysis was not feasible due to the variation in the methods as applied in the included studies.*

In rows 128-130 – The sentence is not clear. Also, please provide more details on how the tool to evaluate quantitative studies formultae an opinion of the study validity and applicability. Also, it is a similar tool to all types of quantitative studies (randomised trials and observational studies)?

***Reply*** *:*

*We have revised it and added these sentences in rows 137-142 :*

*For quantitative studies, a scoring checklist was used to assess the quality of the research hypothesis, the study population, selection bias, exposure, outcome, and confounding; and to formulate an overall opinion of the study's validity and applicability through the accumulation score of the assessment of the method, sampling, data, result, ethics, implications and usefulness of the study. This assessment was used for all papers that have been reviewed, which all have observational study designs.*

--------------------
**Results**
Reviewer #1: Yes, analysis is done as per plan

Reviewer #2:
-Does the analysis presented match the analysis plan?
Yes.
-Are the results clearly and completely presented?
No. The analysis plan is illogical.
-Are the figures (Tables, Images) of sufficient quality for clarity?
No. Figures 2. is confusing.

***Reply :***

*As explained above in our responses about the methods section, we have revised the Macro levels factors into Structural factors and Micro levels factors into Health service factors to avoid confusing the readers. We did a narrative analysis in this systematic review to describe all the health care factors related to case detection delay, not only restricted to specific factors. We used the PRISMA guideline, and the protocol has been registered in the PROSPERO as mentioned in rows 132-133 :*

*The methods and results have been reported following the PRISMA guidelines (see Table S1).*

*-Rows 94-97 :*

*The protocol for this review was based on the Preferred Reporting Items for Systematic Reviews and Meta-Analyses (PRISMA) guidelines for systematic reviews and meta-analyses [14].* *The study protocol was registered in the International Prospective Register of Systematic Reviews (PROSPERO) under reference code CRD42020189274.*

*Figure 2 describes the relation of the factors to case detection delay systematically in three parts: Structural factors, Health service factors, and Intermediate factors. In Figure 2, we also use arrows to indicate the relations between the factors, either as mentioned in the included studies or as inferred.*

Reviewer #3: Figure 1 (flowchart) must provide the reasons for excludion and the number of articles excluded by each reason.

***Reply :***

*It has been revised; we now added the numbers and reasons for exclusion of records. Please see the revised Figure 1.*

In table 1, in the last column that says Sample size (responde rate), I suggest removing response rate as that means something else. If the authors do not know how many individuals were targeted and how many actually were interviewed, qit is impossible to know the responde rate.

***Reply :***
*We have now removed the response rate ( Please see table 1)*

Figure 2 is very good but it is unclear, for example, how number of leprosry cases could lead to descentralization. Also, it is unclear what prejudice among healthcare professionals mean – its prejudice of healthcare professionals towards patients?

***Reply :***

*We have now revised Figure 2 and explained it in the method and results comments above. I hope our explanation in Figure 2 is sufficient.*

*In Figure 2 we only indicate the pathway or relation of the factors to case detection delay, but provide no details.*

*For detailed information of the number of leprosy cases that could lead to decentralisation we now have added information* *in rows 193-196:*

*Decentralisation led people with leprosy to visit health services closer to their home* *with percentages increasing from 23·5% to 46·6% (p<0.001), which led in turn to* *an increase in self-reporting from 38·7% to 51·1% (p=0.010) and to* *a reductions of G2D leprosy at diagnosis from 16.2% to 7.6% (p=0.012).*

*Prejudice among health workers about leprosy was explicitly mentioned (35). It might also concern leprosy patients, but we are not sure. We now explain this in* *rows 223-224:*

*The same study also reported that prejudice about leprosy among basic healthcare professionals often led to unnecessary referrals and thereby delays [35].*

Row 162-163 – information repeated from methods section
***Reply :***

*We removed the repeated information ( Please see row 171)*

Rows 171-176 – it is confusing the definition of macrolevel factors that seem to act as sometimes barriers and sometimes facilitators. I think reframing the names so to go all to barriers or all to facilitators will clarify the interpretation of the text and figure 2.
***Reply :***

*We have changed the Macrolevel terms as indicated in our explanation above.*

*We now only describe the barriers and facilitators in the Structural factors, and not in the Health service factors and Intermediate factors.*

*The information of the barriers and facilitator has been written in* *rows 186-196 :*

*Two studies in Peru and Brasil [30, 35], both published in 2020, reported delays related to poor geographical access to healthcare services. Another barrier described by one study post-elimination in Myanmar (2020) was related to lack of financial and logistic support, which limited community-based leprosy field activities [36]; health staff faced high transportation costs for supervising the leprosy program, and relied on other health programs for field activities [36]. One study in Peru (2010) that evaluated pre and post the leprosy program decentralisation reported that the decentralisation of leprosy control and its integration into primary healthcare was a facilitator that led to significantly earlier case detection (p=0.012) [20]. Decentralisation led people with leprosy to visit health services closer to their home with percentages increasing from 23·5% to 46·6% (p<0.001), which led in turn to an increase in self-reporting from 38·7% to 51·1% (p=0.010) and to a reductions of G2D leprosy at diagnosis from 16.2% to 7.6% (p=0.012).*

*Although generally decentralization of leprosy services is a facilitator, if it is disrupted by factors such as high turnover of health care staff and low commitment, detection delay may occur. We now describe this in* *rows 196-199:*

*In contrast, disruptions of decentralised leprosy care, such as high turnover of health care staff and low commitment, contributed to longer case-detection intervals [35], as reported in a study in Brasil (2020) that aimed to analyse assistance by healthcare management as provided to people affected by leprosy.*

Rows 178-193 – this a nice description of studies but lacks more data on the studies (e.g., country, when it was published and in which context) to improve interpretability of findings. For instance, when the authors say that "the low endemicity of leprosy can also contribute rto delays…", this was done in the entire country of India or in a certain area? Which type of study was that?
***Reply :***

*We have extended the data on the studies*

*-Rows 186-193:*

*Two studies in Peru and Brasil [30, 35] both published in 2020 reported delays related to poor geographical access to healthcare services. Another barrier described by one study post-elimination in Myanmar (2020) was related to lack of financial and logistic support, which limited community-based leprosy field activities [36]; health staff faced high transportation costs for supervising the leprosy program, and relied on other health programs for field activities [36]. One study in Peru (2010) that evaluated pre and post the leprosy program decentralisation reported that the decentralisation of leprosy control and its integration into primary healthcare was a facilitator that led to significantly earlier case detection (p=0.012)*

*-Rows 196-199:*

*In contrast, disruptions of decentralised leprosy care, such as a high turnover of health staff and low commitment, contributed to longer case-detection intervals [35], as reported in one study in Brasil (2020) that aimed to analyse assistance provided to people affected leprosy by healthcare management.*

*-Rows 202-206 :*

*The low endemicity of leprosy can also contribute to delays in detection: one qualitative study with mix method from India that was done in three districts in one state [31] reported increased detection delays and a lack of involvement by health staff in the National Leprosy Elimination Program (NLEP) after leprosy was declared to have been “eliminated” or “controlled” by the central government.*

In row 195-197 – The sentence "Fig 2 includes a further macrolevel factor: health-service organisation and management. Although this umbrella term was not mentioned explicitly in the articles reviewed, there are many allusions to it." Seems lost in the text. Maybe the authors could explain in the methods that the process of aggregating studies in macrolevels when that was not present was a subjective decision made by the authors. Also, what was performed if the authors classification into levels (macro, micro and intermediate levels) was discordant with the original study?
***Reply :***

*We have revised Figure 2 and the term Macro level factors. Please see our explanation about Figure 2 above.*

*We used the causal pathway as described in the included studies to categorise the factors in three groups : Structural factors, Health service factors and Intermediate factors.*

*To make this method more clear, the description has been revised* *in rows 125-127:*

*These variables were then categorised into three main groups: 1) Structural factors; 2) Health service factors; and 3) Intermediate factors by considering causal pathways written in each study reviewed.*

In rows 233-235 – It is not possible to perform a matanalysis on the role of case-finding methods and with delayed case detection? It seems 3 studies can be combined into a polled analysis.

***Reply :***

*Indeed, unfortunately it is not possible to perform a meta-analysis due to studies using different detection methods . Some studies only provided the significance value without providing statistic values such as OR.*

In rows 250-251 – Similar. It is not possible to perform a matanalysis on misdiagnosis was on leprosy late case detection? In addition, what was the most type of misdiagnosys cited?
***Reply :***

*Yes, it was not possible to perform a meta-analysis due to only one study providing an OR with significance value. The other study only provided the statement that misdiagnosis contributed to delay but provided no statistical value.*

*The type of misdiagnosis most cited was arthritis, photosensitivity, nerve disease or other skin diseases. We now added this information* *in rows 264-265:*

*The misdiagnosis that was reported most was arthritis, photosensitivity, nerve disease or other skin diseases.*

In rows 268-270 – What is the causal pathway that the number of examinations
or consultations in healthcare services can delay diagnosys? Maybe a better interpretation is needed. This a signal of bad trained doctors? Lack of laboratories? Or something else? There is any more information associated to the type of clinic (more or less specialised of the first visits, duration of consultations, etc) that could be associated with delays?

***Reply :***

*The delays can happen if there is a long period between the leprosy patient having the first signs or symptoms and the moment of diagnosis as a leprosy case. This problem can be caused by health care services needing several examinations or consultations before being clear that the patient has leprosy. The more visits to the health service patients need before getting diagnosed with leprosy, the longer the delay.*

*As shown in figure 2, first visiting general health services, private health services, and other than skin clinics can contribute to detection delay. So, we conclude that the type of health care services patients first go to also may contribute to case detection delay.*

--------------------
**Conclusions**
Reviewer #1: Conclusions are supported by the data presented

Reviewer #2: -Are the conclusions supported by the data presented?
No. For example, the decentralisation and integration of healthcare services (classified as facilitators) has both advantages and disadvantages.
***Reply :***

*Thank you for the suggestions to look again at the part “the decentralisation and integration of healthcare services” in the Conclusion. Now, we have removed the part about decentralisation and integration of health care from the conclusion section.*

-Are the limitations of analysis clearly described?
Yes.
-Do the authors discuss how these data can be helpful to advance our understanding of the topic under study?
Yes.
-Is public health relevance addressed?
Yes.

Reviewer #3:

In rows 333-334- - the authors cite "leprosy programs could adopt approaches developed by tuberculosis programs, such as a public private mixed (PPM) approach". Please provide minimum details so people can understand which "approach" is that. Many readers could not be familiar with either leprosy or TB.
***Reply :***

*TB and leprosy programs have similar approaches to controlling the transmission of these diseases in the community: to find the case as early as possible to be treated. TB has used PPM as the method to find the case early. So, the Leprosy programs can learn lessons from that program.*

*We have revised and added some information as written* *in rows 345-351:*

*As case detection is sometimes delayed by visits to private clinics and by a lack of community activities such as active leprosy case-finding surveys, leprosy programs could adopt approaches developed by tuberculosis programs to find the cases as early as possible, such as a public-private mixed (PPM) approach and the involvement of community health workers in the national leprosy program. The leprosy program can create health care network services in one district/city involving all public and private health facilities of leprosy coordinated by the District/City Health Office* *to increase the leprosy early case detection [49,50]*

Row 352 – It is confusing why the authors are citing this reference in the sentence. The results are similar to study 53?

***Reply :***

*The reference has been removed*

Rows 355-356 – How WHO post-exposure profilaxy is related to the main objective of your study? Please provide more details on eligibility and how this could contribute to reduce leprosy burden.
***Reply*** *:*

*From the current systematic review, we found that active case finding, like household contact survey, can decrease the case detection delay. For a more effective effort to halt leprosy transmission, which the WHO has recommended, such a contact survey can be followed with post-exposure prophylaxis administered to eligible contacts.* *This intervention will engage the leprosy program to make contact with the community, possibly increasing the community's awareness about leprosy.*

*We now have added information in the passage to make it clearer, as written* *in rows 369-374 :*

*An effective and feasible intervention recommended by the WHO for reducing leprosy cases is to follow a contact survey by providing post-exposure prophylaxis to eligible contacts with single-dose rifampicin (SDR). This intervention can prevent delay effectively due to the leprosy program must make contact with the community directly to increase the awareness of the community through health promotion efforts and do active case finding.*

--------------------

**Editorial and Data Presentation Modifications?**
Use this section for editorial suggestions as well as relatively minor modifications of existing data that would enhance clarity. If the only modifications needed are minor and/or editorial, you may wish to recommend "Minor Revision" or "Accept".

Reviewer #1: Accept

Reviewer #2: Minor Revision.

Reviewer #3: (No Response)

--------------------

**Summary and General Comments**
Use this section to provide overall comments, discuss strengths/weaknesses of the study, novelty, significance, general execution and scholarship. You may also include additional comments for the author, including concerns about dual publication, research ethics, or publication ethics. If requesting major revision, please articulate the new experiments that are needed.

Reviewer #1: Overall a good meta analysis article

Reviewer #2: This systematic review is an interesting study addressing on healthcare factors related to delayed detection of leprosy cases. The findings and conclusions are helpful for formulating leprosy control progarams. The method of data analysis need to be clear and logical.

Reviewer #3: I believe this is a very important work, but the methods described suggest that this is more a literature review rather than a systematic review. I suggest to revise PRISMA guideline to improve the methods and results section. The approach to select papers and extract data from them is not very detailed – which type of papers were you looking for in the SR, inclusion and excludion criteria, methods to extract data and approach to summarise data should be all in the methods. As this type of information was not included, readers cannot be sure if all the literature on the topic was included or if led to too much exclusion. In addition, I believe it is a big limitation only including papers in English. Please see detailed comments below:

***Reply*** *:*

*Our study is a systematic review since we systematically searched for data with eligibility criteria from 2000 to 2021 from the five international databases indexed as the world's representative peer-reviewed journals, as stated in the S1 Text (Search strategy). We analysed them with the team (4 authors) to answer the research aim to know the health care services factors (Intervention/Exposure) related to case detection delay (No Compare) to solve the burden of disability in leprosy (Outcome) in a high and low endemic country (Population) as we now make it clearer in rows 88-90:*

*Improved quality of care and interventions to reduce detection delays depend on the systematic identification of healthcare-related factors to reduce leprosy disability in high and low endemic countries, which is what this systematic literature review aimed to achieve*

*In doing so we followed the PRISMA guidelines. In addition, we registered our study protocol in PROSPERO. We now added the detailed information as requested by the reviewer. The manuscript has also added PICO criteria as the including and excluding criteria. We also did the interpretation and presented the results as described in the method and results section. We did not make a meta-analysis, only a descriptive analysis due to the limitation of information and data written in the source paper, which has many variations of the study that was used in this study. The authors realise that they did not have the capacity in other languages except in English, so it has been declared as the limitation in this study.*
--------------------
